# The Expression of Cell Cycle Cyclins in a Human Megakaryoblast Cell Line Exposed to Simulated Microgravity

**DOI:** 10.3390/ijms25126484

**Published:** 2024-06-12

**Authors:** Alisa A. Sokolovskaya, Ekaterina A. Sergeeva, Arkadiy A. Metelkin, Mikhail A. Popov, Irina A. Zakharova, Sergey G. Morozov

**Affiliations:** Department of Molecular and Cellular Pathophysiology, Institute of General Pathology and Pathophysiology, Baltiyskaya Str. 8, 125315 Moscow, Russia; katya96korn@mail.ru (E.A.S.); armetelkin@gmail.com (A.A.M.); popovcardio88@mail.ru (M.A.P.); izaharova_zia@mail.ru (I.A.Z.); biopharm@list.ru (S.G.M.)

**Keywords:** microgravity, random positioning machine, megakaryocytes, cell cycle, cyclins

## Abstract

The study of the physiological and pathophysiological processes under extreme conditions facilitates a better understanding of the state of a healthy organism and can also shed light on the pathogenesis of diseases. In recent years, it has become evident that gravitational stress affects both the whole organism and individual cells. We have previously demonstrated that simulated microgravity inhibits proliferation, induces apoptosis, changes morphology, and alters the surface marker expression of megakaryoblast cell line MEG-01. In the present work, we investigate the expression of cell cycle cyclins in MEG-01 cells. We performed several experiments for 24 h, 72 h, 96 h and 168 h. Flow cytometry and Western blot analysis demonstrated that the main change in the levels of cyclins expression occurs under conditions of simulated microgravity after 96 h. Thus, the level of cyclin A expression showed an increase in the RPM group during the first 4 days, followed by a decrease, which, together with the peak of cyclin D, may indicate inhibition of the cell cycle in the G2 phase, before mitosis. In addition, based on the data obtained by PCR analysis, we were also able to see that both cyclin A and cyclin B expression showed a peak at 72 h, followed by a gradual decrease at 96 h. STED microscopy data also confirmed that the main change in cyclin expression of MEG-01 cells occurs at 96 h, under simulated microgravity conditions, compared to static control. These results suggested that the cell cycle disruption induced by RPM-simulated microgravity in MEG-01 cells may be associated with the altered expression of the main regulators of the cell cycle. Thus, these data implicate the development of cellular stress in MEG-01 cells, which may be important for proliferating human cells exposed to microgravity in real space.

## 1. Introduction

Gravity negatively affects the human body as a whole and at the cellular level. It is essential to understand what happens during the transformation of both normal and tumor cells. The study of the physiological and pathophysiological processes under extreme conditions contributes to a better understanding of the state of a healthy organism and can also shed light on the pathogenesis of diseases [1,2]. Special attention is paid to modern research investigating the effects of microgravity at the cellular level, including cell morphology, proliferation apoptosis, and gene expression [3,4,5,6].

Effects seen in real microgravity were reproduced with good agreement on a random positioning machine (RPM) [7]. The RPM has been used to study leukemic cells, malignant glioma, and thyroid carcinoma [8,9,10].

Platelets in microgravity have been studied using various experimental models [11]. It has been reported that in in vivo experiments, parabolic flight induced thrombocytopenia in mice [12].

There is evidence that microgravity affects platelet function. This study is supported by space experiments on Discovery STS 51-C and STS 61-C, in which platelets remained monodispersed and showed no activity under microgravity conditions compared to platelets on the ground [13].

Microgravity causes hemorrhagic complications by reducing platelet counts and impairing platelet function. Suping Li, et al. reported that platelet function is suppressed in microgravity and activated in high gravity, revealing the pathogenesis of hemorrhagic and thrombotic diseases associated with changes in gravity [14].

On the other hand, microgravity causes upper body blood stasis, vascular endothelial dysfunction and changes in blood volume and viscosity, all events that may contribute to increased incidences of thrombosis [11,15].

The responses of megakaryocytes to reduced gravity and the associated physiological changes in these cells have not been elucidated. Some researchers have reported that microgravity exposure changes the morphological phenotype, cell adhesion, and gene expression and has an impact on cell survival that is highly dependent upon the cell type as well as on the duration of exposure [16,17].

Despite advances in our understanding of the physiological effects of microgravity on cancer cells, the cellular and molecular processes remain to be explored.

We have previously demonstrated that simulated microgravity suppresses proliferation, induces apoptosis, and alters the morphology and expression of MEG-01 cell surface markers [18]. In this study, we aimed at investigating possible cell cycle mechanisms in a human megakaryoblast cell line under microgravity simulations.

## 2. Results 

In order to investigate the expression of cell cycle cyclins in MEG-01 cells, we performed several experiments for 24 h, 72 h, 96 h and 168 h. First, we investigated the expression of cyclins in MEG-01 megakaryoblast cells by flow cytometry.

**Figure 1:**
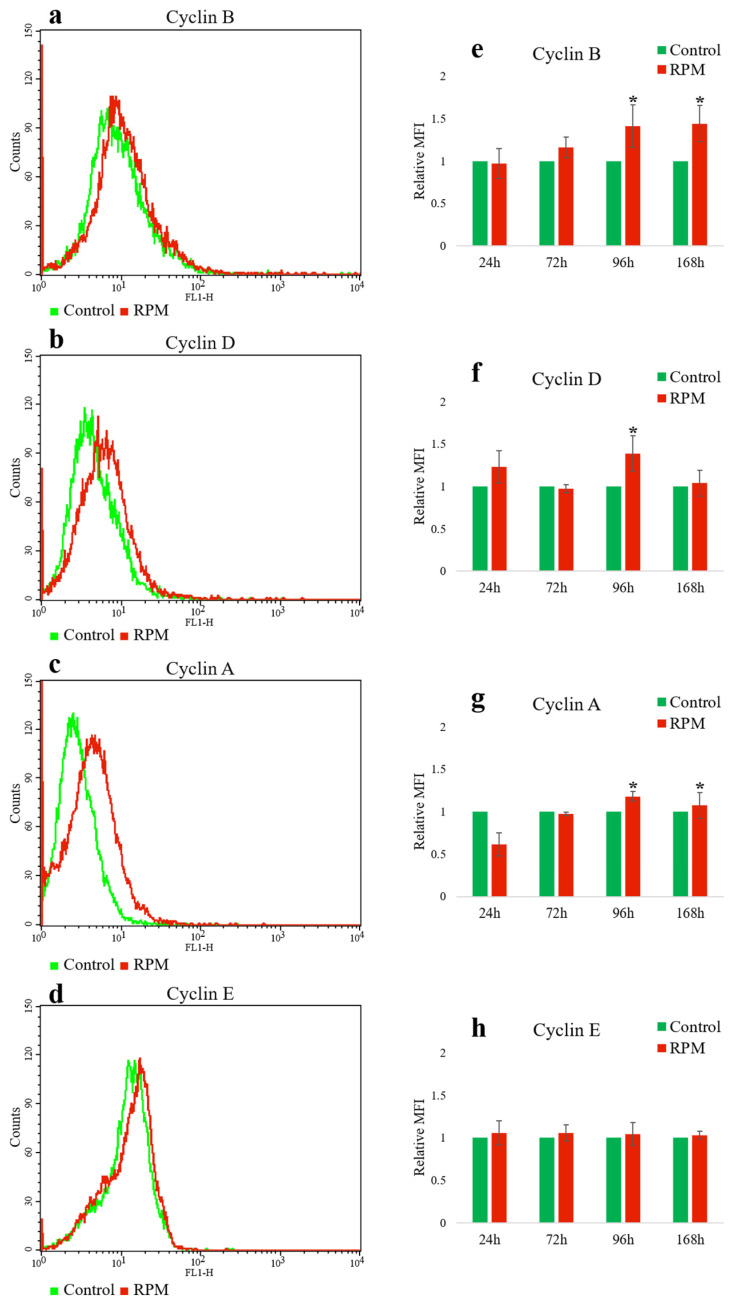
The effect of RPM-simulated microgravity on the expression of cyclins in MEG-01 cells. The flasks with cells in a static position (1 g) were used as controls. Representative histograms (**a**–**d**) and graphs (**e**–**h**) demonstrate the results. Relative mean fluorescence intensity (MFI) in the RPM group measured by flow cytometry was expressed as fold change compared to the control group collected at each corresponding time point. Data are presented as mean ± standard deviation. * *p* < 0.05 compared to static control (*n* = 5).

In our data, flow cytometry analysis showed changes in the expression of all cyclins in MEG-01 megakaryoblast cells. Figure 1a–d show the expression of cyclins as histograms on the left side of the graph.

Correspondingly on the right side of Figure 1e–h, plots are shown with statistical mean, respectively, for the control (1 g) and RPM-modeled microgravity group at different time intervals. The most pronounced changes occur at 96 h in RPM-modeled microgravity compared to the control group (cyclin B: 1.41 ± 0.25 vs. 1; *n* = 3; *p* < 0.05; cyclin A: 1.18 ± 0.06 vs. 1; *n* = 3; *p* < 0.05).

Then, we examined the expression levels of cell cycle cyclins in MEG-01 cells using Western blotting.

Our Western blot data confirmed that the major change in cyclin expression of MEG-01 cells occurred after 96 h under simulated microgravity conditions, compared to the statistical control.

As can be seen in Figure 1, there was an increase in cyclin A expression in the RPM group during the first 4 days, followed by a decrease. There were no particularly pronounced differences in cyclin E expression between the RPM groups compared to the control group (1 g).

Cyclin B expression in MEG-01 cells was most pronounced in the RPM group at 96 h, as measured both by flow cytometry and Western blot.

**Figure 2:**
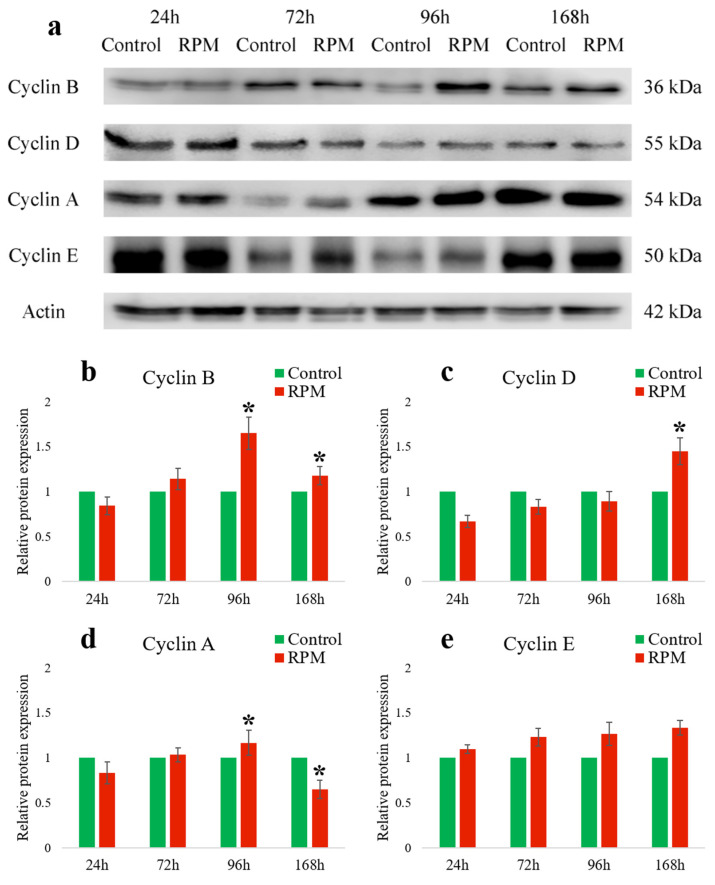
The effects of RPM-simulated microgravity on the expression of cyclins in MEG-01 cells. Representative Western blots (**a**) and their relative band intensity histograms (**b**–**e**) are shown. The flasks with cells in a static position (1 g) were used as controls. Relative protein expression measured by the Western blot method was expressed as fold change compared to the control group collected at each corresponding time point. Data are presented as mean ± standard deviation. * *p* < 0.05 compared to static control (*n* = 3).

The results of our studies show that under the influence of microgravity, MEG-01 cells showed equal levels of cyclins D and E compared to the control group. However, the levels of cyclins A and B increased until they reached their peaks at 96 h (cyclin B: 1.65 ± 0.18 vs. 1; *n* = 3; *p* < 0.05; cyclin A: 1.16 ± 0.14 vs. 1; *n* = 3; *p* < 0.05) (Figure 2).

Furthermore, the quantity of these cyclins decreased at the last time point of 168 h compared to the previous time point and control group (cyclin B: 1.18 ± 0.1 vs. 1; *n* = 3; *p* < 0.05; cyclin A: 0.65 ± 0.1 vs. 1; *n* = 3; *p* < 0.05).

In the next step, we analyzed the effects of RPM-modeled microgravity on cell cycle gene expression in MEG-01 cells.

**Figure 3:**
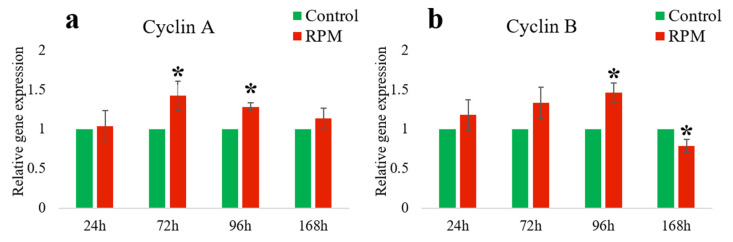
Relative gene expression of cell cycle proteins after exposure to RPM. Relative gene expression (**a**—cyclin A, **b**—cyclin B) measured by the qPCR method was expressed as fold change compared to the control group collected at each corresponding time point. * *p* < 0.05, (*n* = 4).

In contrast, cyclins responsible for distinct phases of the cell cycle showed increased expression, but at different time points: cyclin A RNA showed a peak at 72 h (1.42 ± 0.19 vs. 1; *n* = 3; *p* < 0.05) followed by a gradual decrease at 96 h (1.28 ± 0.05 vs. 1; *n* = 3; *p* < 0.05). Cyclin B RNA peaked at 96 h (1.46 ± 0.12 vs. 1; *n* = 3; *p* < 0.05), followed by a sharp decrease to (0.78 ± 0.08 vs. 1; *n* = 3; *p* < 0.05) compared to the control cells (Figure 3).

**Figure 4:**
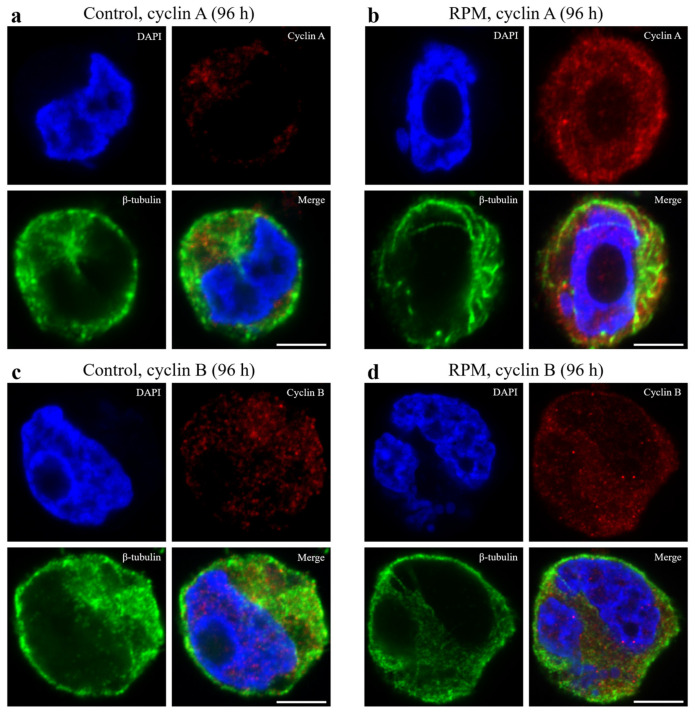
Fluorescence triple staining of MEG-01 cell preparation. Figures (**a**,**c**) are static control group; (**b**,**d**) are RPM group. Nuclei staining with DAPI dye (4’,6-diamidino-2-phenylindole)—blue fluorescent dye; anti-β-tubulin Alexa Fluor 488—green fluorescent dye; and antibodies to cyclin A2 and cyclin B1—red fluorescent dye. Image analysis on an inverted biological microscope Nikon Eclipse Ti2 (Nikon Instruments, Melville, NY, USA). The scale bar is 5 µm.

In addition to these measurements, we performed immunofluorescence studies to investigate the effect of simulated microgravity on cyclin expression. The expression of cyclins A and B was most pronounced. Data obtained by spontaneous emission suppression STED microscopy using a Nikon microscope confirmed that the major change in cyclin expression of MEG-01 cells occurred after 96 h, under simulated microgravity conditions, compared to static controls (Figure 4).

The immunocytochemical staining of β-tubulin-containing structures showed differences between the expression of β-tubulin in the control group and the RPM group of MEG-01 cells after 96 h. In our study, cells under simulated microgravity conditions showed a decrease in β-tubulin expression in the RPM group after 96 h.

We also found that β-tubulin accumulated near the cytoplasmic membrane in the cells under microgravity conditions, while gathering around cell nuclei in the control group.

## 3. Discussion

The effect of weightlessness on apoptosis and cell proliferation depends on different conditions and cell types. Many publications describe changes in the cell cycle of cancer cells under conditions of real and simulated microgravity [19,20,21,22].

It has been shown that the proliferative potential of certain cancer cells, K562, MCF-7, and A549, decreased under simulated microgravity conditions [23,24,25].

Studies have demonstrated that microgravity inhibited cell growth via down-regulation of cell cycle-regulating proteins, such as cyclins B1 and D1 in breast and colorectal cancer cells [24,25]. Rotating wall vessel (RWV) cultured bone marrow CD34+ cells, which appeared to be hindered in their ability to progress through the cell cycle at the S phase, also showed reduced expression of cyclin A, cyclin B, and p21 and demonstrated no alteration in the expression of cyclins D1, D2, D3, and cyclin E, p27, and CDK-2 [26]. Another group demonstrated a reduced expression of cell cycle genes in lymphocytes exposed to the RWV. Authors observed the downregulation of pro-apoptotic genes, and they suggested that extended exposure to simulated microgravity may result in a reduction in the cells’ ability to undergo apoptosis [27].

Studies have proved that microgravity can inhibit the proliferation of Chang Liver Cells (CCL-13) by downregulating cell cycle-related proteins, including cyclin A1 and A2, cyclin D1 and cyclin-dependent kinase 6 [28].

We have recently demonstrated that simulated microgravity inhibits proliferation, induces apoptosis, changes morphology, and alters the surface marker expression of MEG-01 cells. The simulated microgravity impaired the cell cycle development of MEG-01 cells. Analysis of proliferation using intracellular marker Ki-67 and flow cytometry showed a decrease in cell proliferation in the RPM group compared to the static control group after 72 h [18].

In the present study, we investigated the expression of MEG-01 cell cyclins by flow cytometry and Western blot. Both methods together demonstrated that the major change in cyclin expression levels occurred under simulated microgravity conditions after 96 h. Thus, the expression level of cyclin A showed an increase in the RPM group during the first 4 days, followed by a decrease, which, together with the peak in cyclin D, may indicate cell cycle inhibition in the G2 phase, before mitosis. It should also be noted that our results correlate with those obtained by other authors.

The results of several experiments aimed at studying the cell cycle in simulated microgravity were obtained using different cell lines. Consistent with the changes to cell cycle distribution, the levels of intercellular cyclins in K562 cells in rotary cell culture system changed at 12 h, including a decrease in cyclin A and an increase in cyclins B, D, and E, and then began to readjust to control levels. The increases in cyclins D and E transitioned the cells into the S phase, and the decrease in cyclin A led to an accumulation of cells in the S phase [23].

The cell cycle process in DLD-1 cells was markedly affected with reduced viability, reduced colony forming ability, an apoptotic population and dysregulation of cell cycle genes, oncogenes, and cancer progression and prognostic markers [29].

The increased expression of cyclin B is consistent with the increased percentage of cells in the G2/M phase, as cyclin B reaches its maximum during mitosis but must be degraded in a calcium-dependent manner to enter anaphase [30]. The up-regulation of cyclin B during the G2/M phase has been shown to reflect the observation that calcium-mediated gene expression is suppressed as a result of reduced gravitational force [31].

Modelled microgravity caused partial arrest of the G1 phase in rat pheochromocytoma PC12 cells [32]. In addition, both normal vascular smooth muscle cells of mouse and non-neoplastic human breast cancer cells were induced to partial arrest in G2/M 1A (*CDKN1A*) under simulated microgravity [33].

As reported in one study, altered cell morphology, decreased cell viability and an abnormal cell cycle profile were observed in colorectal cancer cell line DLD-1 and lymphoblastic leukemia cell lines in microgravity compared to their static controls. During the cell cycle, a whole series of changes were detected in DLD-1 cells—a decrease in viability and ability to form colonies, signs of dysregulation of cell cycle genes, the presence of oncogenes, markers of cancer progression and prognostic markers [34].

Simultaneous exposure of human fibroblasts to simulated microgravity and radiation resulted in a greater number of chromosomal aberrations than in cells exposed to radiation alone. Expression of genes suppressing the cell cycle (*ABL1* and *CDKN1A*) decreased, while expression of genes promoting the cell cycle (*CCNB1*, *CCND1*, *KPNA2*, *MCM4*, *MKI67*, and *STMN1*) increased under microgravity conditions after carbon ion irradiation [35].

Cyclin gene expression levels significantly affected cancer progression and metastasis, as they can direct cell proliferation or apoptosis. *CDK1* expression was decreased in MOLT-4 and increased in DLD-1 (by five-fold compared to static controls). Expression of genes fundamental to cancer development and progression, including oncogenes and potential cancer stem cell markers, was impaired in microgravity [34].

In addition, based on the data obtained by PCR analysis, we could also see that both cyclin A and cyclin B expression showed peaks at 72 h, followed by a gradual decrease at 96 h. STED microscopy data also confirmed that the main change in cyclin expression of MEG-01 cells occurred after 96 h, under simulated microgravity conditions, compared to static control.

We demonstrated that simulated microgravity delays cell cycle progression of MEG-01 cells, as reflected by changes in the expression of cell cycle cyclins.

## 4. Materials and Methods 

### 4.1. Cell Culture and Culture Conditions

The human megakaryoblast cell line MEG-01 was obtained from the German Collection of Microorganisms and Cell Cultures (DSMZ, Braunschweig, Germany). The cell lines were maintained in RPMI 1640 (Life Technologies, Gaithersburg, MD, USA) supplemented with 10% fetal bovine serum (FBS, Life Technologies) and 1% penicillin/streptomycin solution (Life Technologies) at 37 °C in 5% CO_2_ (Sanyo, Tokyo, Japan).

### 4.2. Random Positioning Machine (RPM)

Microgravity conditions were simulated using Desktop Random Positioning Machine (RPM) (Dutch Space, The Company Astrium EADS, Leiden, The Netherlands) [7].

The flasks with MEG-01 cells were seeded completely filled with culture medium to avoid air bubbles and minimize liquid flow.

The flasks were fixed onto the Desktop RPM at the center of the platform, which was rotated at a speed of 60°/s. The RPM was placed in the culture incubator at 37 °C with 5% CO_2_. The control (static) flasks were placed in the same culture incubator.

### 4.3. Flow Analysis of Cyclins Expression

The samples were fixed under different gravitational conditions with 100% ice-cold methanol for cyclin B detection and 70% ice-cold ethanol for detection of cyclins A, D, and E and stored at −20 °C for at least 12–18 h prior to staining. Samples were stained with anti-cyclin antibodies (BD Biosciences, Franklin Lakes, NJ, USA) according to the manufacturer’s protocol. FITC mouse IgG1 was used as an isotype control to exclude fluorescent background noise. All samples were analyzed using flow cytometry (Becton Dickinson, Franklin Lakes, NJ, USA). At least 15,000 events were acquired for each experimental sample. Results were expressed as MFI.

### 4.4. Western Blotting

Cells were lysed with Radio Immuno Precipitation Assay (RIPA) buffer, mixed with Laemmli sample buffer (4×) and boiled. Proteins were subjected to 12% SDS-PAGE and electroblotted onto Bio-Rad 0.45 μM nitrocellulose membrane (Bio-Rad Laboratories, Hercules, CA, USA). Membranes were blocked by 5% milk (blotting-grade blocker; Bio-Rad Laboratories, Hercules, CA, USA) in TBST buffer at RT for 1 h and then treated overnight at 4 °C (BD Biosciences) with working dilutions of the respective anti-cyclin antibodies in the presence of 5% milk with 0.06% NaN3 in TBST buffer. After that, the membranes were washed with TBST and were then incubated with secondary horseradish peroxidase-conjugated goat antimouse immunoglobulin G (1:1000 dilution; BD Biosciences, USA) at RT for 1 h. A mouse monoclonal anti-actin Ab-5 antibody (1:1000 dilution; BD Biosciences, USA) was used as a loading control.

The detection of bands was implemented using the Odyssey XF Imaging System —LI-COR Biosciences image station (LI-COR Biosciences, Lincoln, NE, Lincoln, NE, USA) and Amersham ECL Western blotting detection kit (GE Healthcare, Chicago, IL, USA) according to the manufacturer’s instructions.

### 4.5. Immunofluorescence Microscopy

For immunostaining, the cells were washed in PBS, fixed in 4% paraformaldehyde for 10 min, and permeabilized with 0.5% saponin for 15 min according to the antibody protocol. After washing with PBS, the cells were incubated in blocking buffer (10% FBS) for 2 h.

Following a further wash with PBS, the cells were incubated with anti-β-tubulin Alexa Fluor 488 (BD Biosciences, USA) together with polyclonal antibodies to cyclins A2 and B1 (AbClonal, Woburn, MA, USA, all antibodies at a dilution of 1:100) overnight at 4 °C. The next day, the cells were washed three times with PBS and incubated with the secondary Goat anti-rabbit Chromeo 546 (AbClonal, USA, all antibodies at a dilution of 1:100) at RT for 1 h in the dark. Cells were washed again three times with PBS and incubated for 15 min with DAPI (4’,6-diamidino-2-phenylindole) (BD Biosciences, USA) at RT. Then, the cells were washed three times with PBS and mounted on glass slides using a mounting medium (Thermo Fisher Scientific, Waltham, MA, USA). The slides were sealed with cover glass and allowed to dry for 2 h before visualization.

At least 20 different microscopic fields were observed for each sample. STED microscopy images showed the linear, striated condensates. Scale bars: 5 µm. The experiment was performed three times with similar results. The presented cells were representative of the respective populations. Widefield images were acquired on an inverted Eclipse Ti2-E microscope (Nikon, Tokyo, Japan), equipped with STEDYCON (Abberior, Göttingen, Germany), Iris 15 Scientific sCMOS camera (Teledyne Photometrics, Tucson, AZ, USA), LED microscope illuminator pE-300^white^ (CoolLED, Andover, UK), Nikon Plan APO 100×/1.45 objective for STED microscopy and CFI S Plan Fluor LWD ADM 20XC objective for fluorescence microscopy. Images were processed in ImageJ version 1.54g.

### 4.6. mRNA Isolation and Real-Time PCR Amplification

The cell samples were washed with phosphate buffer from the culture medium, transferred to PCR tubes in the amount of 1 × 10^6^ cells/mL, and precipitated at 1500 rpm for 5 min. Total RNA was isolated by adding ExtractRNA lysing reagent (Eurogen, Moscow, Russia) according to the manufacturer’s instructions. A 500 μL suspension was mixed by pipetting and left at −80 °C.

After thawing the samples in ExtractRNA (Eurogen, Russia), nucleic acids were isolated by phenol-chloroform extraction and further treated with 70% ethanol. The concentration of nucleic acids and purification efficiency were evaluated using a Nanodrop 2000 spectrophotometer (Thermo Fisher Scientific, Waltham, MA, USA).

The sample was purified from DNA using DNAase (Thermo Fisher Scientific, Waltham, MA, USA); then, cDNA was synthesized using MMLV RT kit (Eurogen, Russia).

The amplification reaction was performed using primers and qPCRmix-HS SYBR (Eurogen, Russia) on a Bio-Rad CFX-96 instrument (Bio-Rad Laboratories, Hercules, CA, USA). The results were processed in Bio-Rad CFX Manager 3.1 and Microsoft Excel 2019 programs using the 2^−ΔΔCt^ method [36].

The primers were as follows (Table 1):

### 4.7. Statistics

The sample was normally distributed and evaluated using the Shapiro–Wilk test. Differences between groups were assessed using one-way analysis of variance. *p* < 0.05 values were considered significant. The analysis was performed using StatSoft Statistica 12 for Windows.

## 5. Conclusions 

We suggest that simulated microgravity delays cell cycle progression of MEG-01 cells, as reflected by changes in the expression of cell cycle cyclins. Thus, these data implicate the development of cellular stress in MEG-01 cells, which may be important for proliferating human cells exposed to microgravity in real space.

Future studies will focus on the molecular mechanisms of different cell behaviors in different cells under simulated microgravity conditions, which will help identify factors that regulate cell death or proliferation.

The obtained data may form the basis for further study of hematopoiesis under weightlessness conditions. At the same time, the obtained data may be in demand in clinical practice for the development of strategies for the prevention of hemostasis disorders in astronauts.

## Figures and Tables

**Table 1:** Sequences of the primers used in PCR.

Gene	Forward Sequence	Reverse Sequence
*GAPDH*	5′-GGAGCGAGATCCCTCCAAAAT-3′	5′-GGCTGTTGTCATACTTCTCATGG-3′
*CCNA2*	5′-CTCTACACAGTCACGGGACAAAG-3′	5′-CTGTGGTGCTTTGAGGTAGGTC-3′
*CCNB1*	5′-TTGGTGTCACTGCCATGTTT-3′	5′-CCGACCCAGACCAAAGTTTA-3′
*CCND1*	5′-GCTGCGAAGTGGAAACCATC-3′	5′-CCTCCTTCTGCACACATTTGA-3′
*CCNE1*	5′-TGTGTCCTGGATGTTGACTGCC-3′	5′-CTCTATGTCGCACCACTGATACC-3′

## Data Availability

The original contributions presented in the study are included in the article, further inquiries can be directed to the corresponding author.

## References

[1] Demontis G.C., Germani M.M., Caiani E.G., Barravecchia I., Passino C., Angeloni D. (2017). Human Pathophysiological Adaptations to the Space Environment. Front. Physiol..

[2] Grimm D., Hemmersbach R. (2022). Translation from Microgravity Research to Earth Application. Int. J. Mol. Sci..

[3] Bizzarri M., Masiello M.G., Giuliani A., Cucina A. (2018). Gravity Constraints Drive Biological Systems toward Specific Organization Patterns: Commitment of cell specification is constrained by physical cues. Bioessays.

[4] Bradbury P., Wu H., Choi J.U., Rowan A.E., Zhang H., Poole K., Lauko J., Chou J. (2020). Modeling the Impact of Microgravity at the Cellular Level: Implications for Human Disease. Front. Cell Dev. Biol..

[5] Prasad B., Grimm D., Strauch S.M., Erzinger G.S., Corydon T.J., Lebert M., Magnusson N.E., Infanger M., Richter P., Krüger M. (2020). Influence of Microgravity on Apoptosis in Cells, Tissues, and Other Systems In Vivo and In Vitro. Int. J. Mol. Sci..

[6] Corydon T.J., Schulz H., Richter P., Strauch S.M., Böhmer M., Ricciardi D.A., Wehland M., Krüger M., Erzinger G.S., Lebert M. (2023). Current Knowledge about the Impact of Microgravity on Gene Regulation. Cells.

[7] Borst A.G., van Loon J.J.W.A. (2009). Technology and Developments for the Random Positioning Machine, RPM. Microgravity Sci. Technol..

[8] Cuccarolo P., Barbieri F., Sancandi M., Viaggi S., Degan P. (2010). Differential behaviour of normal, transformed and Fanconi’s anemia lymphoblastoid cells to modeled microgravity. J. Biomed. Sci..

[9] Becker J.L., Souza G.R. (2013). Using space-based investigations to inform cancer research on Earth. Nat. Rev. Cancer.

[10] Warnke E., Kopp S., Wehland M., Hemmersbach R., Bauer J., Pietsch J., Infanger M., Grimm D. (2016). Thyroid cells exposed to simulated microgravity conditions—Comparison of the fast rotating clinostat and the Random Positioning Machine. Microgravity Sci. Technol..

[11] Locatelli L., Colciago A., Castiglioni S., Maier J.A. (2021). Platelets in Wound Healing: What Happens in Space?. Front. Bioeng. Biotechnol..

[12] Fuse A., Aoki Y., Sato T., Sunohara M., Takeoka H. (2002). Decreased Platelet Level in Peripheral Blood of Mice by Microgravity. Biol. Sci. Space.

[13] Dintenfass L. (1989). Experiment on “discovery” STS 51-C: Aggregation of red cells and thrombocytes in heart disease, hyperlipidaemia and other conditions. Adv. Space Res..

[14] Li S., Shi Q., Liu G., Zhang W., Wang Z., Wang Y., Dai K. (2010). Mechanism of platelet functional changes and effects of anti-platelet agents on in vivo hemostasis under different gravity conditions. J. Appl. Physiol..

[15] Limper U., Tank J., Ahnert T., Maegele M., Grottke O., Hein M., Jordan J. (2021). The Thrombotic Risk of Spaceflight: Has a Serious Problem Been Overlooked for More Than Half of a century?. Eur. Heart J..

[16] Dai K., Wang Y., Yan R., Shi Q., Wang Z., Yuan Y., Cheng H., Li S., Fan Y., Zhuang F. (2009). Effects of microgravity and hypergravity on platelet functions. Thromb. Haemost..

[17] Saxena R., Pan G., McDonald J.M. (2007). Osteoblast and osteoclast differentiation in modeled microgravity. Ann. N. Y. Acad. Sci..

[18] Sokolovskaya A.A., Korneeva E.A., Kolesov D.V., Moskovtsev A.A., Kubatiev A.A. (2019). Inhibition of cell cycle progression and changes in surface markers in MEG-01 megakaryoblastic cells exposed to the random positioning machine. Microgravity Sci. Technol..

[19] Arun R.P., Sivanesan D., Vidyasekar P., Verma R.S. (2017). PTEN/FOXO3/AKT pathway regulates cell death and mediates morphogenetic differentiation of Colorectal Cancer Cells under Simulated Microgravity. Sci Rep..

[20] Chen L., Yang X., Cui X., Zhang Y., Luo X. (2015). Adrenomedullin is a key Protein Mediating Rotary Cell Culture System that Induces the Effects of Simulated Microgravity on Human Breast Cancer Cells. Microgravity Sci. Technol..

[21] Chen Z.Y., Guo S., Li B.B., Jiang N., Li A., Yan H.F., Yang H.M., Zhou J.L., Li C.L., Cui Y. (2019). Effect of Weightlessness on the 3D Structure Formation and Physiologic Function of Human Cancer Cells. BioMed Res. Int..

[22] Cortés-Sánchez J.L., Callant J., Krüger M., Sahana J., Kraus A., Baselet B., Infanger M., Baatout S., Grimm D. (2021). Cancer Studies under Space Conditions: Finding Answers Abroad. Biomedicines.

[23] Yi Z., Xia B., Xue M., Zhang G., Wang H., Zhou H., Sun Y., Zhuang F. (2009). Simulated microgravity inhibits the proliferation of K562 erythroleukemia cells but does not result in apoptosis. Adv. Space Res..

[24] Kopp S., Slumstrup L., Corydon T.J., Sahana J., Aleshcheva G., Islam T., Magnusson N.E., Wehland M., Bauer J., Infanger M. (2016). Identifications of novel mechanisms in breast cancer cells involving duct-like multicellular spheroid formation after exposure to the Random Positioning Machine. Sci. Rep..

[25] Chang D., Xu H., Guo Y., Jiang X., Liu Y., Li K., Pan C., Yuan M., Wang J., Li T. (2013). Simulated microgravity alters the metastatic potential of a human lung adenocarcinoma cell line. In Vitro Cell. Dev. Biol..

[26] Plett P.A., Frankovitz S.M., Abonour R., Orschell-Traycoff C.M. (2009). Proliferation of human hematopoietic bone marrow cells in simulated microgravity. In Vitro Cell. Dev. Biol..

[27] Morabito C., Lanuti P., Caprara G.A., Marchisio M., Bizzarri M., Guarnieri S., Mariggiò M.A. (2019). Physiological Responses of Jurkat Lymphocytes to Simulated Microgravity Conditions. Int. J. Mol. Sci..

[28] Ho C.N.Q., Tran M.T., Doan C.C., Hoang S.N., Tran D.H., Le L.T. (2021). Simulated Microgravity Inhibits the Proliferation of Chang Liver Cells by Attenuation of the Major Cell Cycle Regulators and Cytoskeletal Proteins. Int. J. Mol. Sci..

[29] Infanger M., Kossmehl P., Shakibaei M., Bauer J., Kossmehl-Zorn S., Cogoli A., Curcio F., Oksche A., Wehland M., Kreutz R. (2006). Simulated weightlessness changes the cytoskeleton and extracellular matrix proteins in papillary thyroid carcinoma cells. Cell Tissue Res..

[30] Santella L., Ercolano E., Nusco G.A. (2005). The cell cycle: A new entry in the field of Ca2+ signaling. Cell. Mol. Life Sci..

[31] Echard A., O’Farrell P.H. (2003). The degradation of two mitotic cyclins contributes to the timing of cytokinesis. Curr. Biol..

[32] Wang J., Zhang J., Bai S., Wang G., Mu L., Sun B. (2009). Simulated microgravity promotes cellular senescence via oxidant stress in rat PC12 cells. Neurochem. Int..

[33] Coinu R., Chiaviello A., Galleri G., Franconi F., Crescenzi E., Palumbo G. (2006). Exposure to modeled microgravity induces metabolic idleness in malignant human MCF-7 and normal murine VSMC cells. FEBS Lett..

[34] Vidyasekar P., Shyamsunder P., Arun R., Santhakumar R., Kapadia N.K., Kumar R., Verma R.S. (2015). Genome Wide Expression Profiling of Cancer Cell Lines Cultured in Microgravity Reveals Significant Dysregulation of Cell Cycle and MicroRNA Gene Networks. PLoS ONE.

[35] Ikeda H., Muratani M., Hidema J., Hada M., Fujiwara K., Souda H., Yoshida Y., Takahashi A. (2019). Expression Profile of Cell Cycle-Related Genes in Human Fibroblasts Exposed Simultaneously to Radiation and Simulated Microgravity. Int. J. Mol. Sci..

[36] Livak K.J., Schmittgen T.D. (2001). Analysis of relative gene expression data using real-time quantitative PCR and the 2^−ΔΔCT^ Method. Methods.

